# Real-world renal function among patients with multiple myeloma in the United States

**DOI:** 10.1038/s41408-021-00492-6

**Published:** 2021-05-21

**Authors:** Joseph Mikhael, Erin Singh, Megan S. Rice

**Affiliations:** 1 Applied Cancer Research and Drug Discovery, Translational Genomics Research Institute, City of Hope Cancer Center, Phoenix, AZ 85004 USA; 2 Sanofi, Cambridge, MA 02142 USA

**Keywords:** Myeloma, Combination drug therapy

Dear Editor,

Multiple myeloma (MM) is the second most common hematologic malignancy and is associated with substantial patient burden^1^. Despite the introduction of newer agents, patients with MM continue to experience relapses and/or become treatment refractory^2^. Renal impairment has been shown to affect up to 50% of patients with MM^3^, and has been reported to be an independent predictor of poor survival outcomes, with a median survival of approximately half that of MM patients without renal impairment^4,5^.

The aims of this analysis were to assess change in renal function by drug class (i.e., proteasome inhibitor [PI], immunomodulatory drug [IMiD], and monoclonal antibody [mAb]) among patients with MM and renal impairment (defined as estimated glomerular filtration rate [eGFR] < 50 mL/min/1.73 m^2^ using the Modification of Diet in Renal Disease equation [MDRD]) in early treatment lines, and to assess real-world patient outcomes by baseline renal status, renal response, and drug class.

This study used the nationwide Flatiron Health electronic health record-derived de-identified database. The Flatiron Health database is a longitudinal database, comprising de-identified patient-level structured and unstructured data, curated via technology-enabled abstraction^6,7^. During the study period, the de-identified data originated from ~280 cancer clinics (~800 sites of care). The study included 10,389 patients diagnosed with MM from January 1, 2011, to November 30, 2019. After excluding patients with probable missing treatment information, there were 6994 patients who received ≥1 line of MM therapy and had information on race. Another four patients with unlikely creatinine levels (i.e., extremely high or low) were excluded, resulting in 6990 patients (Supplemental Fig. 1).

eGFR-MDRD was calculated from creatinine lab values using the following equation: 175 × (creatinine mg/dL)^−1.154^ × (age)^−0.203^ × (0.742 if female) × (1.212 if Black/African American)^8–10^. The distribution of patients was assessed by eGFR-MDRD level (<50 and ≥50 mL/min/1.73 m^2^) at the start of first- (1 L) and second-line (2 L) therapy. Overall survival (OS) was evaluated by treatment line, stratified by eGFR-MDRD level at the start of the treatment line. Renal response was assessed in patients with eGFR-MDRD < 50 mL/min/1.73 m^2^ at the start of treatment, who had ≥1 eGFR-MDRD measurement during the treatment line; using International Myeloma Working Group recommendations, patients with complete renal response (CRR) were defined as patients with ≥1 eGFR-MDRD measurement ≥60 mL/min/1.73 m^2^ during the treatment line.

Logistic regression models were used to examine the association between treatment class and CRR status. These models were adjusted for the following clinically relevant variables, as they were potential confounders of the examined associations: other treatment classes received, age, sex, race, practice type, year of therapy line, and cytogenetic risk. Kaplan–Meier analyses and Cox proportional hazard models were used to examine OS from the start of 1 L therapy and 2 L therapy by baseline renal status and by both treatment class and renal response. Cox proportional hazard models were adjusted, as described above. The data that support the findings of this study have been originated by Flatiron Health, Inc. These de-identified data may be made available upon request, and are subject to a license agreement with Flatiron Health; interested researchers should contact DataAccess@flatiron.com to determine licensing terms. Flatiron Health, Inc, did not participate in the analysis of this data.

Patient characteristics overall and by eGFR-MDRD level (<50 and ≥50 mL/min/1.73 m^2^) at the start of 1 L therapy are shown in Supplemental Table 1. The mean age at start of initial therapy was 68.3 years, 45.8% of patients were female, and 17.2% were Black/African American. One quarter (*n* = 1722) of patients at the start of initial therapy had eGFR-MDRD < 50 mL/min/1.73 m^2^ (including 10.5% with eGFR-MDRD < 30 mL/min/1.73 m^2^), approximately half (*n* = 3465) had eGFR-MDRD ≥ 50 mL/min/1.73 m^2^, and the remainder had unknown levels. On average, patients with eGFR-MDRD < 50 mL/min/1.73 m^2^ at the start of initial therapy were older (71.3 vs 67.3 years) and more likely to be diagnosed at stage III (37.7% vs 10.3%) compared with patients with eGFR-MDRD ≥ 50 mL/min/1.73 m^2^.

From the start of 1 L and 2 L therapy, patients with eGFR-MDRD < 50 mL/min/1.73 m^2^ had worse OS compared with those with eGFR-MDRD ≥ 50 mL/min/1.73 m^2^ (1 L median OS, 3.46 vs 5.89 years; 1 L unadjusted hazard ratio [HR], 1.79 [95% confidence interval (CI), 1.63–1.97]; 2 L median OS, 2.67 vs 4.44 years; 2 L unadjusted HR, 1.70 [95% CI, 1.52–1.91]; Supplemental Fig. 2). These differences remained statistically significant after adjustment for several factors, including age (1 L multivariable-adjusted hazard ratio [aHR], 1.58 [95% CI, 1.43–1.73]; 2 L multivariable aHR, 1.49 [95% CI, 1.33–1.68]; Supplemental Table 2). Similar results were seen for patients with eGFR-MDRD < 30 mL/min/1.73 m^2^ compared with those with eGFR-MDRD ≥ 50 mL/min/1.73 m^2^ from the start of 1 L (multivariable aHR, 2.14; 95% CI, 1.90–2.41) and 2 L therapy (multivariable aHR, 1.95; 95% CI, 1.66–2.29).

Among all patients with eGFR-MDRD < 50 mL/min/1.73 m^2^ at the start of the treatment line and ≥1 eGFR-MDRD measurement during the treatment line (*N* = 1682 in 1 L and 920 in 2 L), 38.5% of patients achieved a CRR during 1 L therapy, but only 19% achieved a CRR during 2 L therapy. The majority of these patients with renal impairment had 1 L and 2 L use of PI (77% and 64%, respectively). These patients were significantly more likely to have a CRR compared with those without PI use in both 1 L (adjusted odds ratio [aOR], 1.36; 95% CI, 1.04–1.77) and 2 L therapy (aOR, 2.09; 95% CI, 1.38–3.16) in multivariable-adjusted models.

Approximately half of patients with renal impairment had IMiD use in 1 L and 2 L therapy. In both treatment lines, patients with IMiD use were significantly more likely to have a CRR compared with those without IMiD use in multivariable models, particularly in 1 L therapy (aOR, 2.14; 95% CI, 1.68–2.72).

When we examined PI and IMiD use in combination together among patients with renal impairment, 36% of patients had combination PI and IMiD treatment, 42% used PIs but not IMiDs, 14% used IMiDs but not PIs, and 8% used neither in 1 L therapy. In 2 L, 26% of patients had combination PI and IMiD treatment, 38% used PIs but not IMiDs, 26% used IMiDs but not PIs, and 10% used neither. In 1 L and 2 L therapy, patients with both PI and IMiD use were significantly more likely to have a CRR compared with those without use of either treatment class (1 L aOR, 2.35 [95% CI, 1.54–3.60]; 2 L aOR, 3.89 [95% CI, 1.71–8.86]). In 2 L therapy, patients with PI use but no IMiD use also were significantly more likely to have a CRR compared with those without use of either (aOR, 2.54; 95% CI, 1.15–5.59; Fig. 1). Patients with eGFR-MDRD < 30 mL/min/1.73 m^2^ with both PI and IMiD use were significantly more likely to have a CRR compared with those without the use of either treatment class in 1 L (aOR, 5.46; 95% CI, 1.77–16.8), but there was no association in 2 L (aOR, 0.50; 95% CI, 0.06–4.17).

**Fig. 1:**
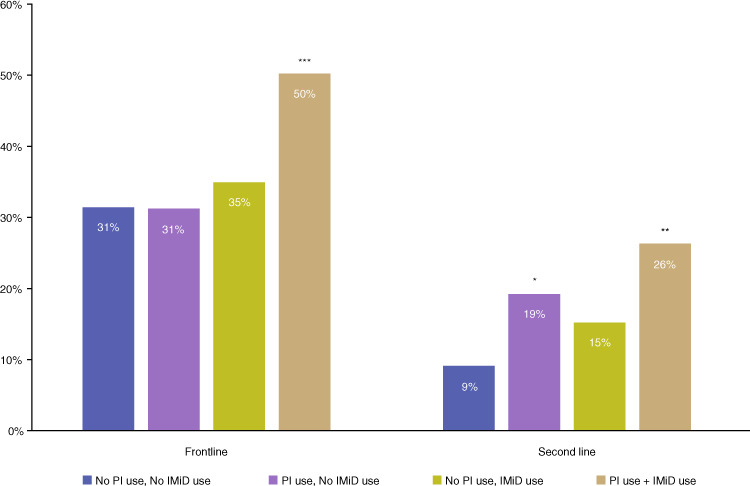
Complete renal response (CRR) by combined proteasome inhibitor (PI) use and immunomodulatory drug (IMiD) use in frontline and second line. Logistic regression models were used to examine the association between treatment class (i.e., PI and IMiD) and CRR status adjusted for other treatment classes received, age, sex, race, practice type, year of therapy line, and cytogenetic risk. *P < 0.05, **P < 0.01, ***P < 0.001 vs no PI or IMiD use from multivariable models. Multivariable models adjusted for other treatment classes received, age, sex, race, practice type, year of therapy line, and cytogenetic risk.

Low mAb use in early treatment lines during the database time frame (through November 2019) prevented most analyses of this treatment class. Few patients (<1%) were treated with mAb frontline and ~10% had mAb use as 2 L therapy. We did not observe a significant difference in CRR by mAb use in 2 L (22% vs 19%; aOR, 1.51; 95% CI, 0.84–2.74). Due to small sample sizes, we were unable to examine mAb combination therapies.

Patients receiving combination PI and IMiD treatment who had a CRR had significantly better OS compared with patients without use of either treatment, who did not have a CRR in both frontline (aHR, 0.52; 95% CI, 0.37–0.73) and second line (aHR, 0.53; 95% CI, 0.32–0.88; Supplemental Table 3). This relationship was not observed for patients with eGFR-MDRD < 30 mL/min/1.73 m^2^; however, due to the relatively smaller sample size, we cannot make definitive conclusions for this subgroup.

In this study, MM patients with renal impairment were more likely to be older and to present with ISS stage III at diagnosis, which has been reported in a previous phase III study^11^. Patients with renal impairment exhibited decreased OS compared with non-renally impaired patients. Patients treated with combination therapy that included PIs and IMiDs together in early treatment lines were more likely to have a CRR, supporting the known benefits of triplet vs doublet therapy. In addition, OS was prolonged among patients with PI and IMiD combination therapy who achieved a CRR, highlighting the benefits of both combination therapy and renal response. Moreover, these data underscore the importance of providing patients the best therapy for their MM.

This study has some limitations. We were unable to discern whether renal impairment was due to MM or other causes. Though we adjusted for several baseline characteristics, including age and practice type, we may not have fully accounted for factors influencing receipt of triplet regimens. In addition, as few patients received mAbs or had very low renal function (eGFR-MDRD < 30 mL/min/1.73 m^2^) in early treatment lines, power for analyses in these subgroups was limited. The primary strengths of this study are the use of repeated eGFR-MDRD assessments and adjustment for several potential confounders.

MM patients with renal insufficiency have been shown to have poorer outcomes and continue to have unmet medical need. Overall, these data suggest that using combination therapy early with the goal of inducing a renal response in these patients is not only feasible, but also may result in improved outcomes. Future investigations with larger datasets that include newer agents may improve the understanding of the optimal combination treatment regimens for these patients.

## Supplementary information

Supplemental Materials
