# Supplementary material for: Real-world renal function among patients with multiple myeloma in the United States
Source: Blood Cancer J. 2021 May 21;11(5):99. doi: 10.1038/s41408-021-00492-6 (PMC8140071; doi:10.1038/s41408-021-00492-6)
Supplement: Supplementary file 1 — Supplemental Materials [file 41408_2021_492_MOESM1_ESM.docx]

**Supplemental materials**

**Fig S1. Flatiron MM cohort patients.**


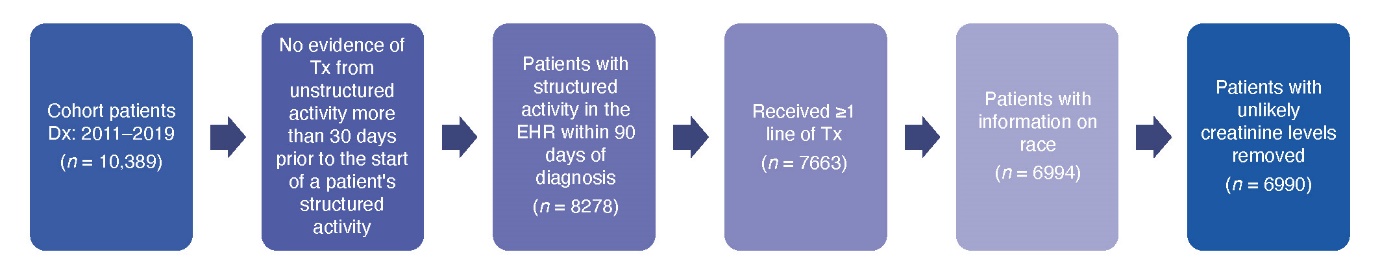


Dx, diagnosis; EHR, electronic health record; MM, multiple myeloma; Tx, treatment.

**Fig S2. Kaplan-Meier curves**

**
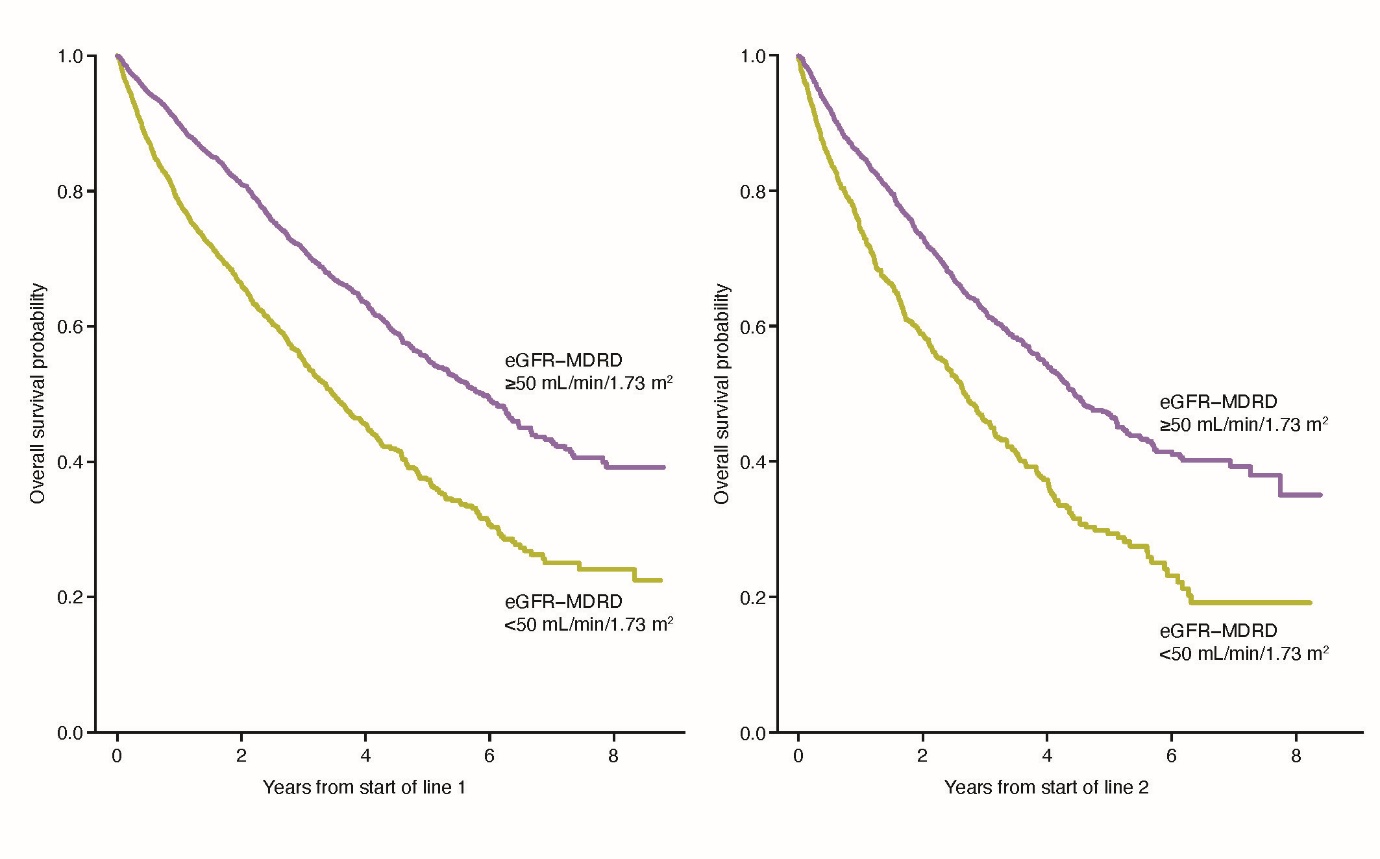
** eGFR, estimated glomerular filtration rate; MDRD, Modification of Diet in Renal Disease equation.

**Table S1. Patient characteristics/disposition by eGFR-MDRD at start of initial therapy**

|  | All patients  (*N* = 6990) | eGFR-MDRD  <50 mL/min/1.73 m^2^  (*n* = 1772) | eGFR-MDRD  ≥50 mL/min/1.73 m^2^  (*n* = 3465) |
| --- | --- | --- | --- |
| Age at start of first-line therapy, years |  |  |  |
| Mean (SD); median | 68.3 (10.5); 70.0 | 71.3 (9.6); 73.0 | 67.3 (10.5), 68.0 |
| Female, % | 45.8 | 47.6 | 45.2 |
| Race, % |  |  |  |
| Asian | 1.9 | 2.0 | 1.8 |
| Black/African American | 17.2 | 15.1 | 18.9 |
| White | 68.0 | 70.9 | 67.5 |
| Other | 12.9 | 12.0 | 11.8 |
| Practice type, % |  |  |  |
| Academic | 10.4 | 9.6 | 10.9 |
| Community | 89.6 | 90.4 | 89.1 |
| ISS stage at diagnosis, % |  |  |  |
| Stage I | 19.3 | 5.2 | 30.6 |
| Stage II | 18.8 | 15.7 | 23.1 |
| Stage III | 18.7 | 37.7 | 10.3 |
| Unknown/not documented | 43.3 | 41.4 | 36.0 |

eGFR, estimated glomerular filtration rate; ISS, International Staging System; MDRD, Modification of Diet in Renal Disease equation; SD, standard deviation.

**Table S2. Overall survival by eGFR-MDRD level at the start of frontline and second-line therapy**

|  | | Patients, *n* | Deaths, *n* | | | Median OS, years | Unadjusted hazard ratio (95% CI) | | | Age-adjusted hazard ratio (95% CI) | Multivariable-adjusted* hazard ratio (95% CI) | | |
| --- | --- | --- | --- | --- | --- | --- | --- | --- | --- | --- | --- | --- | --- |
| First line | |  |  | | |  |  | | |  |  |  |  |
| eGFR-MDRD  ≥50 mL/min/1.73 m^2^ | 3465 | | | 1009 | 5.89 | | | 1 (Ref) | 1 (Ref) | | | 1 (Ref) |  |
| eGFR-MDRD  <50 mL/min/1.73 m^2^ | 1772 | | | 795 | 3.46 | | | 1.79  (1.63–1.97) | 1.56  (1.42–1.71) | | | 1.58  (1.43–1.73) |  |
| Second line |  | | |  |  | | |  |  | | |  |  |
| eGFR-MDRD  ≥50 mL/min/1.73 m^2^ | 2319 | | | 782 | 4.44 | | | 1 (Ref) | 1 (Ref) | | | 1 (Ref) |  |
| eGFR-MDRD  <50 mL/min/1.73 m^2^ | 969 | | | 469 | 2.67 | | | 1.70  (1.52–1.91) | 1.46  (1.30–1.64) | | | 1.49  (1.33–1.68) |  |

CI, confidence interval; eGFR, estimated glomerular filtration rate; MDRD, Modification of Diet in Renal Disease equation; OS, overall survival.

* Adjusted for other treatment classes received, age, sex, race, practice type, year of therapy line, and cytogenetic risk.

**Table S3. Overall survival by PI use, IMiD use, and CRR in frontline and second line**

| **Treatment line** | **Treatment and responder status** | **Patients, *n*** | **Deaths, *N*** | **Median OS, years** | **Unadjusted hazard ratio  (95% CI)** | **Age-adjusted hazard ratio (95% CI)** | **Multivariable-adjusted* hazard ratio (95% CI)** |
| --- | --- | --- | --- | --- | --- | --- | --- |
| Frontline | No PI use, No IMiD use, and non-responder | 96 | 64 | 2.93 | 1 (Ref) | 1 (Ref) | 1 (Ref) |
|  | PI use, IMiD use, and non-responder | 298 | 97 | 3.63 | 0.72 (0.53–0.99) | 0.76 (0.55–1.04) | 0.72 (0.52–1.00) |
|  | No PI use, No IMiD use, and responder | 44 | 22 | 4.08 | 0.65 (0.40–1.06) | 0.75 (0.46–1.21) | 0.81 (0.49–1.31) |
|  | PI use, IMiD use, and responder | 300 | 83 | 5.83 | 0.45 (0.33–0.63) | 0.55 (0.39–0.76) | 0.52 (0.37–0.73) |
| Second-line | No PI use, No IMiD use, and non-responder | 80 | 52 | 1.90 | 1 (Ref) | 1 (Ref) | 1 (Ref) |
|  | PI use, IMiD use, and non-responder | 177 | 77 | 2.95 | 0.71 (0.50–1.01) | 0.81 (0.57–1.16) | 0.82 (0.56–1.21) |
|  | No PI use, No IMiD use, and responder | 8 | 3 | Not achieved | 0.89 (0.28–2.84) | 1.03 (0.32–3.31) | 0.97 (0.30–3.19) |
|  | PI use, IMiD use, and responder | 63 | 24 | 3.94 | 0.52 (0.32–0.84) | 0.51 (0.32–0.83) | 0.53 (0.32–0.88) |

CI, confidence interval; CRR, complete renal response; HR, hazard ratio; IMiD, immunomodulatory drug; PI, proteasome inhibitor.

* Adjusted for other treatment classes received, age, sex, race, practice type, year of therapy line, and cytogenetic risk.
